# Dietary intake, lifestyle behaviors, and sleep architecture among night-shift medical residents: a cross-sectional study

**DOI:** 10.3389/fnut.2026.1851443

**Published:** 2026-06-08

**Authors:** Maya Yusri Rousan, Hadeel Ali Ghazzawi, Khaled Al Oweidat, Renad Ali Alawamleh

**Affiliations:** 1 Department of Nutrition and Food Technology, School of Agriculture, The University of Jordan, Aljubeiha, Jordan; 2 Department of Internal Medicine, School of Medicine, The University of Jordan, Amman, Jordan

**Keywords:** dietary intake, junk food consumption, medical residents, night-shift work, sleep quality

## Abstract

**Background:**

Medical residents are at risk of sleep disturbances due to extended working hours, rotating night shifts, elevated occupational stress, and irregular meal schedules. Although evidence suggests that diet and lifestyle may influence sleep quality and sleep architecture, these relationships remain insufficiently explored among medical residents.

**Objective:**

This study aimed to assess dietary intake, lifestyle, physical activity levels, and sleep quality and architecture among medical residents, and to investigate the links between sleep quality and other measures.

**Methods:**

This cross-sectional study included 50 medical residents, aged 24–34 years recruited from JUH, Amman, Jordan. Dietary intake was assessed using 3-day food records and analyzed for total energy consumption, macronutrient, fiber, magnesium, and zinc intake. The IPAQ was used to measure physical activity. While the PSQI was used to measure sleep quality, along with objective sleep parameters, including total sleep duration, light sleep, deep sleep, REM sleep, and nocturnal awakenings, were measured using a wearable sleep-tracking device over the same 3-day period. Spearman correlation and multivariable linear regression analyses were performed, with statistical significance set at *p* < 0.05.

**Results:**

Zinc intake was positively associated with deep sleep duration (*r* = 0.434, *p* = 0.010). Higher total energy intake (*r* = 0.367, *p* = 0.030), carbohydrate intake (*r* = 0.413, *p* = 0.014), and dietary fiber intake (*r* = 0.428, *p* = 0.010) were positively associated with nocturnal awakenings. In multivariable regression models, more frequent junk food consumption independently predicted longer total sleep duration (*β* = 19.22, *p* = 0.026) and longer light sleep duration (*β* = 13.45, *p* = 0.042). No significant independent associations were observed between lifestyle factors and PSQI global score.

**Conclusion:**

Dietary intake and lifestyle behaviors appear to be important correlates of sleep duration and sleep architecture among medical residents. Zinc intake was associated with longer deep sleep duration, whereas higher energy, carbohydrate, and fiber intake were associated with greater sleep fragmentation. Frequent junk food consumption was associated with longer sleep duration but also greater light sleep, suggesting poorer sleep architecture. With the prevalence of sleep disturbances among medical residents, nutritional modification may support sleep quality and occupational wellbeing.

## 1 Introduction

The demanding working hours and disruption of the body’s natural sleep–wake cycle, which are common in night shift medical residents, can significantly affect the health and productivity of healthcare workers. However, the specific connection between lifestyle factors, such as meal composition, meal timing, sleep, and physical activity, and the wellbeing of medical residents is still not fully understood.

Working outside of regular daytime hours, known as shift work, has been associated with a higher risk of metabolic syndrome, this relationship is thought to result from several biological and environmental changes (1). Medical residents, within the framework of the University of Jordan’s Internal Medicine Residency Program, are physicians undertaking a structured 4-year postgraduate training initiative in internal medicine (2).

Epidemiological studies among shift worker population, including medical residents, have demonstrated an association with elevated mortality rates, alongside a spectrum of health conditions, including various cancers (3), as well as Alzheimer’s disease and dementia (4, 5), cardiovascular disease (6), and diabetes (7). Nutritional status, physical activity, and sleep quality are fundamental factors influencing overall health, productivity, and wellbeing (8). Nutrition is essential for cognitive function, metabolic equilibrium, and energy management (9). Consequently, poor dietary practices, including irregular meal timing or meals lacking essential nutrients, associated with weight gain, increased stress, and psychological difficulties (10). Likewise, physical activity is critical for the prevention of metabolic disorders and the maintenance of overall health (11). Sleep deprivation or compromised sleep quality has been associated with disturbances in metabolic control and a heightened susceptibility to metabolic conditions (12).

Previous studies have highlighted the individual negative impacts of poor nutrition, insufficient sleep, and physical inactivity on health outcomes. However, the combined influence of these factors remains underexplored, especially through objective and quantitative assessments in the medical residency context. To address this gap, the present cross-sectional study used several assessment methods, including 3-day dietary records, sleep data from smartwatches, and validated questionnaires for sleep and physical activity, to examine the relationships of lifestyle factors and sleep related factors among medical residents. Consequently, the primary aim of the study was to evaluate the association between dietary habits, sleep quality, and physical activity levels among night shift medical residents enrolled in the University of Jordan Internal Medicine Residency Program. We hypothesized that unfavorable dietary habits and lower physical activity levels would be associated with poorer subjective and objective sleep outcomes among night-shift medical residents.

## 2 Materials and methods

### 2.1 Study design and participants

This observational cross-sectional study included 50 male and female medical residents aged between 24 and 34 years who regularly worked night shifts. The study aims to investigate the relationship between diet, lifestyle, and sleep stages and quality among medical residents. The study population was recruited from the Jordan University Hospital (JUH) in Amman, Jordan. Department heads who were relevant to the study recommended potential participants who worked either rotational or fixed night shifts. To reduce bias and make sure that the same criteria were used to include and exclude people, an initial screening questionnaire was used. The participants were Jordanian medical residents who were already working regular night shifts (12, 24, or 36 h) as part of their hospital residency programs. Individuals who had diagnosed health problems that are known to affect sleep (like circadian rhythm disorders, sleep apnea, mental health problems, cardiovascular, respiratory, or metabolic disorders) were excluded from the study. Individuals who took sleep aids, herbs, or supplements, or who heavily drank alcohol, were also excluded. Individuals who were interested in the study were briefed on the study’s objectives and procedures, and they had to sign informed consent forms before any data was collected. Data were collected between October and December 2025.

### 2.2 Dietary assessment

Dietary intake was assessed using a 3-day food record (2 working nights days and 1 weekend day). Participants recorded and sent every meal, snack, and beverage consumed through WhatsApp messages, often including photographs before and after consumption along with the corresponding intake time. Detailed instructions regarding dietary recording procedures and portion size estimation were explained to participants during the initial interview and consent form. Mixed dishes were analyzed using standardized recipes and ingredient estimations based on commonly consumed local Jordanian dishes. In cases of missing food photographs or incomplete records, participants were contacted for clarification to improve data completeness and reduce underreporting.

Portion sizes were estimated using household measures, food photographs, and participant clarification when needed. Standardized portion estimation methods were used, including the number of tablespoons consumed, standard package sizes, a matchbox-sized portion (approximately 30 g) for cheese, a palm-sized estimation for meat portions, and a standardized volume of approximately 250 mL for liquid dairy products such as milk and yogurt drinks. Fast-food meals were generally standardized based on commonly available serving sizes, with clarification regarding meal size (e.g., small or large) and the inclusion of beverages when applicable. For packaged food products, participants were additionally asked to provide photographs of the nutrition facts label whenever available.

The collected food data were subsequently analyzed using ESHA Food Processor software (version 10.9.0), utilizing the composition of local Jordanian food dishes (13) to estimate nutrient intake. Variables extracted for the analysis included total energy intake (kcal/day) and macronutrients intake (carbohydrates, protein, and fat), and their percentage contribution to total energy intake.

Additionally, specific dietary components known to influence sleep architecture were included in the analysis, including dietary fiber (g), magnesium (mg), and zinc (mg). These micronutrients were selected due to their established roles in neurotransmitter regulation and circadian rhythm stability, providing a more comprehensive understanding of the diet-sleep relationship among medical residents.

### 2.3 Sleep assessment

#### 2.3.1 Subjective sleep quality (PSQI)

A validated Arabic version of the Pittsburgh Sleep Quality Index (PSQI) was used to assess subjective sleep quality. The PSQI is a widely used, self-administered tool that assesses sleep quality and disturbances over a 1-month period through seven components, including Sleep Latency (time to fall asleep), Sleep Duration (hours slept), Habitual Sleep Efficiency (actual sleep time vs. time in bed), Sleep Disturbances (awakenings, pain, coughing), Use of Sleeping Medication, and Daytime Dysfunction (energy, mood, and alertness issues), plus Subjective Sleep Quality (overall feeling of sleep). To ensure data quality and accuracy, the questionnaire was completed by participants under the direct supervision of the researcher; this allowed participants to seek clarifications in real-time and minimized potential misinterpretation of the items. The PSQI helps clinicians and researchers identify poor sleepers and understand patterns; a global score ranging from 0 to 21, with >5 typically indicating poor sleep quality, with higher scores signaling worse issues (14).

#### 2.3.2 Objective sleep stages and efficiency

Objective sleep data were measured using the Huawei Band 10 smartwatch, a wearable device for sleep monitoring in free-living conditions. Sleep-related parameters, including total sleep duration and wearable-derived sleep stage estimates, were collected over the assessment period. The assessment period covered 3 days in the same period of dietary intake assessment. The sleep variables included total sleep duration (minutes), and the minutes and percentage contribution of each sleep stage to the total sleep duration: deep sleep, rapid eye movement (REM) sleep, light sleep, and nocturnal awaking times.

### 2.4 Physical activity assessment

Physical activity was assessed using a validated Arabic version of the International Physical Activity Questionnaire (IPAQ) Short Form, which assesses the intensity, frequency, and duration of activities over the previous 7 days (15). Under researcher supervision, to ensure clarity and minimize misinterpretation, participants self-reported their engagement in vigorous activity, moderate activity, walking, and sitting. Data were converted into Metabolic Equivalent of Task-minutes per week (MET-min/week) according to the standard IPAQ scoring protocol. Participants were categorized into three physical activity levels: low active (inactive), moderately active, and highly active to examine the role of exercise as a potential confounding factor in sleep quality outcomes.

### 2.5 Lifestyle and health status

Lifestyle behaviors that may be influencing sleep were assessed, including junk food consumption and caffeine intake were assessed using a two-step approach. First, participants responded to direct questions concerning their average weekly frequency of junk food consumption (e.g., burgers, pizza, shawarma, and similar fast-food meals) and daily caffeine intake (including coffee, tea, and energy drinks). Second, these self-reported habits were cross-referenced and verified against the 3-day food records to ensure data accuracy and minimize recall bias.

The junk food variable represented the self-reported weekly frequency of fast-food consumption and was analyzed as a frequency-based behavioral variable rather than a quantitative measure of caloric or nutritional intake. In contrast, total energy and nutrient intake were calculated separately from the 3-day food records using ESHA Food Processor software.

### 2.6 Statistical analysis

All statistical analyses were performed using Jamovi (Version 2.6.44.0; Computer Software, retrieved from https://www.jamovi.org). The normality of continuous variables was assessed using the Shapiro–Wilk test. Because several dietary intake variables and sleep parameters were not normally distributed, Spearman’s rank correlation analyses were conducted to examine the associations between dietary intake variables (including total energy, macronutrients, fiber, and magnesium and zinc) and both objective sleep parameters and subjective sleep quality (PSQI global score). To further evaluate independent associations, multivariable linear regression analyses were executed for specific objective sleep outcomes (PSQI global score, total sleep duration, light sleep duration, and number of awakenings) that exhibited significant correlations in the bivariate analyses. The regression models incorporated essential covariates such as IPAQ score, BMI, total energy intake, frequency of junk food consumption, and daily coffee intake. These variables were selected based on clinical relevance, previous literature, and significant associations observed in the bivariate analyses. Prior to model interpretation, assumptions of linear regression, including linearity, homoscedasticity, normality of residuals, and multicollinearity, were evaluated. Multicollinearity was assessed using variance inflation factor (VIF) values, and no evidence of problematic multicollinearity was detected. For all statistical tests, a two-tailed significance level of *p* < 0.05 was used.

### 2.7 Ethical approval

The Institutional Review Board at the University of Jordan (IRB at UJ) approved the research proposal submitted by Dr. Hadeel Ali Ghazzawi from the School of Agriculture, Decision No. (491/2025). The IRB is located at the Deanship of Scientific Research at The University of Jordan. Participation in the study was entirely voluntary, participants were free to withdraw from the study at any time. Written informed consent was obtained from all participants before data collection, and all collected data were anonymized to protect participant confidentiality.

## 3 Results

### 3.1 Descriptive statistics of the study population

Data from 50 medical residents describing demographic, anthropometric, lifestyle, and sleep characteristics are presented in Table 1. The mean age of participants was 24 ± 2.79 years, with the majority being female (66%). The mean BMI was 24.3 ± 4.08 kg/m^2^, with most participants classified as normal weight (60%), while 40% were overweight or obese. Most participants had a low-risk waist-to-hip ratio (90%). The mean total energy intake was 1796 ± 543 kcal/day. Based on IPAQ classification, most participants had a moderate level of physical activity (62%), followed by low (26%) and high (12%) levels. The mean PSQI global score was 7.82 ± 4.09, indicating overall poor sleep quality. Accordingly, 66% of participants were classified as poor sleepers, while only 34% had good sleep quality.

**Table 1:** Descriptive characteristics for the participants (*n* = 50).

Variable	Value		
Age (years)	24 ± 2.79		
Gender *n* (%)	Female	33	(66.0%)
	Male	17	(34.0%)
BMI (kg/m^2^)	24.3 ± 4.08		
BMI classification *n* (%)	Normal weight	30	(60.0%)
	Overweight	15	(30.0%)
	Obese Class I	4	(8.0%)
	Obese Class II	1	(2.0%)
Waist to hip ratio *n* (%)	High risk	5 (10.0%)	
	Low risk	45 (90.0%)	
Total energy intake (kcal/day)	1796 ± 543		
IPAQ score (min/week)	1,255 (720–2,600)		
IPAQ *n* (%)	High	6	(12.0%)
	Low	13	(26.0%)
	Moderate	31	(62.0%)
PSQI global score	7.82 ± 4.09		
PSQI *n* (%)	Good (≤5)	17	(34.0%)
	Poor (>5)	33	(66.0%)

Data are presented as mean ± standard deviation, median (interquartile range), or number (percentage), as appropriate. BMI, body mass index; min, minutes; IPAQ, International Physical Activity Questionnaire; PSQI, Pittsburgh Sleep Quality Index.

### 3.2 Daily dietary intake profile

Dietary intake data obtained from the 3-day dietary records and analyzed using ESHA software are presented in Table 2. The mean total energy intake was 1796 ± 543 kcal/day. The average carbohydrate, protein, and fat intakes were 212 ± 78.3 g/day, 76.1 ± 31.4 g/day, and 72.0 ± 27.4 g/day, respectively. The median fiber intake was 15.5 g/day (10.6–20.1), while zinc intake was relatively low at 4.67 ± 2.89 mg/day. Magnesium intake showed a median of 116 mg/day (85.4–147).

**Table 2:** Daily dietary intake of the study participants.

Variable	Values
Total energy intake (kcal/day)	1796 ± 543
Carbohydrates (g/day)	212 ± 78.3
Protein (g/day)	76.1 ± 31.4
Fat (g/day)	72.0 ± 27.4
Fiber (g/day)	15.5 (10.6–20.1)
Zinc (mg/day)	4.67 ± 2.89
Magnesium (mg/day)	116 (85.4–147)

Data are presented as mean ± standard deviation or median (interquartile range), as appropriate.

### 3.3 Objective and subjective sleep characteristics

Objective sleep parameters and PSQI global scores are summarized in Table 3. The mean PSQI global score was 7.82 ± 4.09, indicating overall poor sleep quality among participants, with 66% classified as poor sleepers. The mean total sleep duration was 438 ± 93.7 min. Light sleep constituted the largest proportion of sleep (55.2 ± 9.41%), followed by deep sleep (23.8 ± 7.75%) and REM sleep (19.7 ± 6.65%). The mean duration of light, deep, and REM sleep was 246 ± 70.9, 104 ± 39.3, and 86.5 ± 36.7 min, respectively. The median number of awakenings was 1.00 (0–2).

**Table 3:** Sleep characteristics of participants.

Variable	Value		
PSQI global score	7.82 ± 4.09		
PSQI *n* (%)	Good (≤5)	17	(34.0%)
	Poor (>5)	33	(66.0%)
Total sleep duration (min)	438 ± 93.7		
Light sleep (min)	246 ± 70.9		
Light sleep (%)	55.2 ± 9.41		
Deep sleep (min)	104 ± 39.3		
Deep sleep (%)	23.8 ± 7.75		
REM sleep (min)	86.5 ± 36.7		
REM sleep (%)	19.7 ± 6.65		
Awakenings	1.00 (0–2)		

Data are presented as mean ± standard deviation, median (interquartile range), or number (percentage) as appropriate. Min, minutes, PSQI, Pittsburgh Sleep Quality Index; REM, rapid eye movement.

### 3.4 Correlation analysis between nutrition and sleep

Spearman correlation analyses between dietary intake variables and sleep parameters are presented in Table 4. Significant positive correlations were observed between total energy intake (*r* = 0.367, *p* = 0.030), carbohydrate intake (*r* = 0.413, *p* = 0.014), and dietary fiber intake (*r* = 0.428, *p* = 0.010) with the number of awakenings. In addition, zinc intake was positively associated with deep sleep duration (*r* = 0.434, *p* = 0.010). No significant correlations were observed between dietary variables and PSQI global score or total sleep duration.

**Table 4:** Correlations between dietary intake and sleep parameters.

Sleep parameters		Total energy intake	CHO (g)	Protein (g)	Fat (g)	Dietary fiber (g)	Magnesium (mg)	Zinc (mg)
PSQI score	*r*	−0.076	−0.027	−0.135	−0.132	−0.027	−0.135	0.082
	*p*-value	0.664	0.877	0.441	0.451	0.877	0.446	0.646
Total sleep duration (min)	*r*	0.054	0.253	−0.048	−0.214	0.087	0.122	0.109
	*p*-value	0.757	0.143	0.785	0.218	0.618	0.492	0.541
Deep sleep (min)	*r*	0.055	0.081	0.224	−0.037	−0.100	−0.007	0.434*
	*p*-value	0.752	0.642	0.197	0.832	0.569	0.966	0.010
REM (min)	*r*	0.104	0.283	−0.109	−0.054	0.153	0.224	−0.179
	*p*-value	0.552	0.100	0.534	0.758	0.379	0.202	0.312
Light sleep (min)	*r*	0.043	0.230	0.011	−0.223	0.147	0.102	0.143
	*p*-value	0.804	0.183	0.948	0.197	0.400	0.564	0.419
Awakenings	*r*	0.367*	0.413*	0.055	0.292	0.428*	0.187	0.045
	*p*-value	0.030	0.014	0.755	0.089	0.010	0.290	0.802

*p < 0.05, **p < 0.01, ***p < 0.001. *r*, Spearman’s correlation coefficient. CHO, carbohydrates; min, minutes; PSQI, Pittsburgh Sleep Quality Index; REM, rapid eye movement.

### 3.5 Correlation analysis between lifestyle factor and sleep

Spearman correlation analyses were conducted to examine the associations between PSQI global score and lifestyle-related variables, including IPAQ score, BMI, total energy intake, junk food consumption per week, and coffee intake per day. As shown in Table 5, none of the examined variables were significantly associated with PSQI global score (all *p* > 0.05).

**Table 5:** Associations between lifestyle factors and PSQI score.

Lifestyle variables		PSQI score
IPAQ score	*r*	0.237
	*p*-value	0.098
BMI	*r*	−0.152
	*p*-value	0.291
Total energy intake	*r*	−0.126
	*p*-value	0.383
Junk food per week	*r*	−0.074
	*p*-value	0.611
Coffee per day	*r*	0.129
	*p*-value	0.371

*p < 0.05, **p < 0.01, ***p < 0.001. *r*, Spearman’s correlation coefficient; IPAQ, International Physical Activity Questionnaire.

Multivariable linear regression analysis was conducted to examine independent predictors of sleep quality, as assessed by the PSQI global score (Table 6). None of the examined variables, including IPAQ score, BMI, total energy intake, junk food consumption, and coffee intake, were independently associated with PSQI global score (all *p* > 0.05).

**Table 6:** Multivariable linear regression analysis predicting PSQI global score.

Predictor	Estimate	SE	95% confidence interval		*t*	*p*	VIF
			Lower	Upper			
Intercept	9.8478	5.85530	−2.12762	21.82326	1.682	0.103	
IPAQ score (min/week)	8.19e-4	6.70e-4	−5.52e−4	0.00219	1.221	0.232	1.13
BMI	−0.0246	0.19030	−0.41378	0.36464	−0.129	0.898	1.11
Total energy intake	−8.24e−4	0.00132	−0.00353	0.00188	−0.623	0.538	1.11
Junk food	−0.1843	0.40376	−1.01009	0.64148	−0.456	0.651	1.19
Coffee intake	−0.1886	0.64166	−1.50095	1.12373	−0.294	0.771	1.14

*p < 0.05, **p < 0.01, ***p < 0.001. Estimate = *β* (beta coefficient). SE, standard error. *t*, *t*-statistic; *p*, *p*-value; VIF, variance inflation factor; IPAQ, International Physical Activity Questionnaire. Model fit: *R*^2^ = 0.071.

Spearman correlation analyses between lifestyle factors and objective sleep parameters are presented in Table 7. A significant positive correlation was observed between total energy intake and the number of awakenings (*r* = 0.367, *p* = 0.030). Junk food consumption was positively associated with total sleep duration (*r* = 0.457, *p* = 0.006) and light sleep duration (*r* = 0.407, *p* = 0.015). No significant associations were found between IPAQ score, BMI, or coffee intake and the examined sleep parameters (all *p* > 0.05).

**Table 7:** Associations between lifestyle factors and sleep parameters.

Lifestyle variables		Total sleep duration min	Deep sleep (min)	Deep sleep %	REM (min)	REM %	Light sleep (min)	Light sleep %	Awakenings
IPAQ score	*r*	0.005	−0.060	−0.072	0.092	0.147	−0.016	−0.099	0.174
	*p*-value	0.978	0.733	0.683	0.599	0.398	0.928	0.570	0.317
BMI	*r*	0.010	0.151	0.121	0.012	−0.088	0.017	−0.169	0.293
	*p*-value	0.957	0.387	0.487	0.947	0.616	0.924	0.331	0.088
Total energy intake	*r*	0.054	0.055	−0.000	0.104	0.044	0.043	−0.130	0.367*
	*p*-value	0.757	0.752	0.998	0.552	0.801	0.804	0.457	0.030
Junk food	*r*	0.457**	0.157	−0.094	0.316	−0.064	0.407*	−0.028	0.118
	*p*-value	0.006	0.367	0.593	0.064	0.716	0.015	0.872	0.501
Coffee	*r*	−0.111	−0.133	−0.082	−0.186	−0.234	0.044	0.261	−0.048
	*p*-value	0.527	0.447	0.641	0.285	0.175	0.804	0.131	0.785

*p < 0.05, **p < 0.01, ***p < 0.001. *r*, Spearman’s correlation coefficient; IPAQ, International Physical Activity Questionnaire; BMI, body mass index; min, minutes.

To further evaluate the independent effects of lifestyle and dietary factors on objectively measured sleep outcomes, multivariable linear regression analyses were conducted for selected sleep parameters that demonstrated significant associations in correlation analyses. The results are presented in Tables 8–10. Junk food consumption was found to be an independent predictor of total sleep duration (*β* = 19.22, *p* = 0.026) and light sleep duration (*β* = 13.45, *p* = 0.042). In addition, total energy intake was independently associated with increased awakenings during sleep (*β* = 0.000881, *p* = 0.022). No significant associations were observed between IPAQ score, BMI, or coffee intake and the examined sleep outcomes (all *p* > 0.05).

**Table 8:** Multivariable linear regression analysis predicting total sleep duration minutes.

Predictor	Estimate	SE	95% confidence interval		*t*	*p*	VIF
			Lower	Upper			
Intercept	350.53303	119.1313	106.8821	594.1840	2.942	0.006	
IPAQ score (min/week)	−0.00579	0.0136	−0.0337	0.0221	−0.424	0.674	1.13
BMI	3.08975	3.8718	−4.8290	11.0085	0.798	0.431	1.11
Total energy intake	−0.01468	0.0269	−0.0697	0.0403	−0.546	0.589	1.11
Junk food	19.21936	8.2149	2.4180	36.0207	2.340	0.026	1.19
Coffee intake	2.82705	13.0551	−23.8736	29.5277	0.217	0.830	1.14

*p < 0.05, **p < 0.01, ***p < 0.001. Estimate = *β* (beta coefficient). SE, standard error; *t*, *t*-statistic. *p*, *p*-value; VIF, variance inflation factor; IPAQ, International Physical Activity Questionnaire. Model fit: *R*^2^ = 0.170.

**Table 9:** Multivariable linear regression analysis predicting light sleep minutes.

Predictor	Estimate	SE	95% confidence interval		*t*	*p*	VIF
			Lower	Upper			
Intercept	238.53156	91.6740	51.0371	426.0260	2.602	0.014	
IPAQ score (min/week)	−0.00593	0.0105	−0.0274	0.0155	−0.565	0.576	1.13
BMI	−0.30877	2.9795	−6.4024	5.7849	−0.104	0.918	1.11
Total energy intake	−0.01077	0.0207	−0.0531	0.0316	−0.520	0.607	1.11
Junk food	13.45094	6.3215	0.5220	26.3799	2.128	0.042	1.19
Coffee intake	7.71180	10.0462	−12.8349	28.2585	0.768	0.449	1.14

*p < 0.05, **p < 0.01, ***p < 0.001. Estimate = *β* (beta coefficient). SE, standard error; *t*, *t*-statistic; *p*, *p*-value; VIF, variance inflation factor; IPAQ, International Physical Activity Questionnaire. Model fit: *R*^2^ = 0.141.

**Table 10:** Multivariable linear regression analysis predicting number of awakenings during sleep.

Predictor	Estimate	SE	95% confidence interval		*t*	*p*	VIF
			Lower	Upper			
Intercept	−2.6720	1.6118	−5.9685	0.62454	−1.658	0.108	
IPAQ score (min/week)	1.78e-4	1.85e-4	−2.00e-4	5.55e-4	0.963	0.344	1.13
BMI	0.0784	0.0524	−0.0288	0.18549	1.496	0.146	1.11
Total energy intake	8.81e-4	3.64e-4	1.36e-4	0.00162	2.420	0.022	1.11
Junk food	0.0115	0.1111	−0.2158	0.23882	0.103	0.918	1.19
Coffee intake	0.0898	0.1766	−0.2714	0.45108	0.509	0.615	1.14

*p < 0.05, **p < 0.01, ***p < 0.001. Estimate = *β* (beta coefficient). SE, standard error. *t*, *t*-statistic; *p*, *p*-value; VIF, variance inflation factor; IPAQ, International Physical Activity Questionnaire. Model fit: *R*^2^ = 0.244.

Overall, higher total energy, carbohydrate, and dietary fiber intake were positively correlated with increased nocturnal awakenings, while zinc intake was associated with greater deep sleep duration. Additionally, junk food consumption independently predicted longer total sleep duration and increased light sleep duration. On the other hand, the given the relatively small sample size and the number of variables examined, the analyses were considered exploratory and hypothesis-generating.

## 4 Discussion

Recent evidence suggests that certain dietary components may influence sleep architecture, particularly in populations exposed to high physiological and psychological stress, such as medical residents working night shifts. In the present study, wearable-derived sleep measures were obtained using a smartwatch to provide practical estimates of sleep-related parameters in free-living conditions. Although wearable devices provide convenient and practical sleep monitoring in free-living settings, their accuracy in differentiating sleep stages remains lower than polysomnography, particularly for deep and REM sleep measurements. Therefore, the wearable-derived sleep stage findings should be interpreted cautiously. In the present study, the smartwatch was used to provide objective estimates of sleep-related parameters rather than a clinical diagnostic assessment of sleep architecture.

### 4.1 Zinc intake and deep sleep

The current study found that higher zinc intake was associated with prolonged deep sleep duration. This implies that zinc consumption may be related to facilitating deeper, restorative sleep. A possible explanation is that zinc regulates neurotransmitters, including glutamate and gamma-aminobutyric acid (GABA), which are important for sleep, and are known to be modulated by zinc. These neurotransmitters are synthesized and metabolized with the help of zinc. Zinc insufficiency has been linked to changes in glutamate and GABA levels, which may cause sleep disturbances and insomnia, according to studies (16). Zinc also contributes to the synthesis of melatonin, which is essential for the circadian rhythm and sleep cycle. Zinc supplementation has been demonstrated in studies to raise melatonin levels, which may result in longer and better-quality sleep (17). Moreover, zinc deficiency has been linked to dysregulation of growth hormones and other sleep-related hormones, which may exacerbate sleep disorders (18). These results agree with prior studies that associate regular dietary zinc consumption with sleep duration and quality. An analysis of data from the National Health and Nutrition Examination Survey (NHANES) revealed that reduced zinc intake from dietary sources was significantly correlated with diminished sleep duration and heightened sleep disturbances in U.S. adults (19).

### 4.2 Total energy intake and sleep continuity

The present study found that higher total caloric intake was associated with higher awakenings times during sleep. This suggests that higher caloric intake may be linked to impaired sleep continuity. May be explained by several physiological mechanisms. Primarily, a high-caloric load induces a significant postprandial thermic effect, elevating body temperature (20). Scientific evidence suggests that the maintenance of stable sleep requires a progressive decline in body temperature; a malfunction in this cycle of body cooling results in the experience of insomnia, leading to fragmented sleep (21). On the other hand, the metabolic impact of a high dietary glycemic load often results in postprandial hyperglycemia and subsequent compensatory hyperinsulinemia; this glycemic instability following evening meals may be linked to fragmented sleep and diminished sleep quality (22). Collectively, these physiologic responses may be associated with higher wake after sleep onset (WASO) and disrupted sleep continuity. These findings are consistent with previous literature linking high energy intake, particularly late in the evening, to poor sleep stability. For instance, St-Onge et al. (23) suggest that high energy dietary patterns, particularly those high in saturated fat and sugars, may disrupt sleep continuity and reduce restorative sleep stages. Furthermore, Crispim et al. (24) reported that higher food intake close to bedtime was associated with adverse sleep outcomes, including reduced sleep efficiency, prolonged sleep latency, and increased wake after sleep onset, particularly in relation to nocturnal fat intake.

### 4.3 Carbohydrate intake and sleep fragmentation

The study found that higher intake of carbohydrates was associated with higher wake after sleep onset (WASO). This suggests that greater carbohydrate consumption may be linked to distribute sleep continuity. The proposed mechanism involves high glycemic load diets inducing postprandial hyperglycemia followed by compensatory hyperinsulinemia, which may be related to fluctuations in blood glucose levels and activation of counter-regulatory hormones such as epinephrine and cortisol (25). Furthermore, experimental evidence indicates that high-carbohydrate diets can alter sleep architecture by reducing slow-wave sleep and increasing rapid eye movement (REM) sleep. Such changes in sleep structure may result in lighter, less stable sleep, thereby increasing the likelihood of awakenings during the night. These physiological responses may be related to arousal and interfere with sleep maintenance (26). Finally, carbohydrate intake may influence sleep through neurochemical pathways. High-glycemic-index carbohydrate meals have been shown to affect tryptophan availability and serotonin production, which are involved in sleep regulation. Although this mechanism is primarily linked to sleep initiation, alterations in these pathways may also indirectly affect overall sleep stability (27). These findings are consistent with previous literature. In the Gangwisch et al. (22) study, they found that total carbohydrate intake was not independently associated with insomnia prevalence, but they found that a higher dietary glycemic index was associated with significantly higher odds of prevalent insomnia at baseline. Additionally, the Zhao et al. (28) study reported that excessive intake of low-quality carbohydrates was associated with poorer sleep patterns and adverse sleep behaviors, supporting the notion that carbohydrate-related metabolic disturbances may negatively affect sleep continuity.

### 4.4 Dietary fiber intake and nocturnal awakenings

The current study found that higher dietary fiber intake was associated with increased WASO. This suggests that greater fiber intake may influence sleep fragmentation. Although most studies demonstrate a positive association between fiber intake and sleep, this association may have a negative impact when fiber is consumed at night, as observed in the sample of this study. A plausible mechanism may be involves delayed gastric emptying and prolonged gastrointestinal transit time associated with high fiber consumption (29). In addition, fiber increases gastrointestinal-related side effects such as bloating, flatulence, cramping, and constipation (30), which may be associated with higher nocturnal micro-arousals and WASO. The present findings can be contextualized within the existing literature examining dietary fiber and sleep outcomes. Starting with the Zhang et al. (31) study, it was found that higher dietary fiber intake was significantly associated with better subjective sleep quality, as indicated by lower PSQI scores. Interestingly, St-Onge et al. (32) reported that higher dietary fiber intake was associated with reduced stage 1 sleep and increased slow-wave sleep duration, suggesting improved sleep depth. However, sleep continuity parameters such as WASO were not assessed. The present findings indicate that while dietary fiber may support deeper sleep stages and sleep quality, its gastrointestinal effects, particularly in the short term or depending on fiber type and timing, may concurrently increase nocturnal awakenings and sleep fragmentation.

### 4.5 Junk food consumption and sleep-related parameters

The present study found that higher weekly junk food consumption was associated with increased total sleep duration while concurrently being linked to a greater proportion of light sleep stages. This may reflect an association between higher junk food intake and less restorative sleep patterns characterized by a greater proportion of light sleep stages. A plausible mechanism involves the dietary glycemic index affecting blood glucose levels; high glycemic foods (such as junk food) may be related to rapid increases in blood glucose, leading to transient hyperglycemia, which could contribute to sleepiness and potentially disrupt normal sleep patterns (33). The elevated dietary glycemic index and glycemic load, primarily driven by the increased consumption of added sugars and refined grains typical in UPFs, are associated with poor sleep quality (22). The association between high glycemic index and glycemic load diets and related to impaired sleep maintenance and early morning awakening (26). Mehmood et al. (34) reported a significant positive association between junk food consumption and poorer subjective sleep quality, indicating that individuals with worse perceived sleep quality were more likely to engage in unhealthy eating behaviors, including frequent junk food intake. Similarly, Rodríguez et al. (35) demonstrated that excessive consumption of processed and ultra-processed foods was associated with poorer quality of life and impaired sleep quality, including reduced daily sleep duration. Although much of the existing literature has focused on short sleep duration, sleep quality is a multidimensional construct that extends beyond total sleep time. It is also possible that medical residents experiencing greater sleep deprivation consumed more junk food and subsequently slept longer during recovery periods, which may partially explain the observed associations. Furthermore, the wearable-derived estimation of sleep stages should be interpreted cautiously, particularly for light and deep sleep measurements. Overall, the present findings suggest that higher junk food intake may be associated with longer sleep duration accompanied by a greater proportion of light sleep stages, which may reflect less restorative sleep patterns despite longer total sleep duration.

### 4.6 Confounders

Several potential confounding factors should be considered when interpreting the present findings. Although several covariates, including BMI, physical activity, caffeine intake, and total energy intake, were included in the regression models, residual confounding cannot be excluded. Factors such as meal and coffee timing, psychological stress, chronotype, and variability in shift schedules may have influenced both dietary behaviors and sleep-related parameters. In addition, all participants were night-shift medical residents; however, shift duration specialty workload, sleep opportunity and scheduling patterns were not fully standardized across participants. Therefore, the observed associations should be interpreted cautiously.

## 5 Conclusion

This study suggest that dietary intake is associated with objective sleep parameters among Jordanian medical residents. A higher intake of zinc was positively associated with an extended duration of deep sleep, indicating a possible function of this micronutrient in facilitating restorative sleep. On the other hand, higher intake of total energy, carbohydrates and fibres were associated to sleep fragmentation and increases WASO. In addition, frequent junk food consumption was significantly associated to predictor of total sleep duration but also prolonged light sleep, highlighting the influence of dietary patterns on sleep quantity. No significant associations were observed between total energy intake, BMI, coffee intake, or physical activity levels and total sleep duration within this sample. These findings suggest that specific dietary components, rather than overall caloric intake alone, may be associated with sleep-related parameters.

Given the cross-sectional design and moderate sample size, causal relationships cannot be established. However, the results contribute to the growing body of evidence supporting the importance of chrono-nutrition and micronutrient adequacy in sleep health. Future longitudinal and interventional studies are warranted to clarify underlying mechanisms and to determine whether targeted nutritional strategies can improve sleep quality and circadian regulation in young adult populations.

## 6 Limitations

This study has several limitations. The cross-sectional design prevents causal inference. The relatively small sample size may have limited statistical power to detect weaker associations and increase the possibility of type I error and model overfitting. Therefore, the findings should be interpreted cautiously and considered exploratory and hypothesis-generating. Dietary intake, physical activity, and subjective sleep quality were assessed using self-reported tools, which are subject to recall and reporting bias. Additionally, residual confounding from unmeasured factors such as stress, chronotype, and caffeine timing cannot be excluded. Also, temporal mismatch existed between the assessment periods used in the present study. Subjective sleep quality assessed by the PSQI reflected sleep patterns over the previous month, whereas dietary intake and wearable-derived sleep measures were assessed over a shorter 3-day period. Therefore, this inconsistency should be considered when interpreting associations involving PSQI outcomes. Finally, the inclusion of only Jordanian medical residents may limit the generalizability of the findings.

## Data Availability

The raw data supporting the conclusions of this article will be made available by the authors, without undue reservation.
